# Real-World Evidence for the Association between Heat-Related Illness and the Risk of Psychiatric Disorders in Taiwan

**DOI:** 10.3390/ijerph19138087

**Published:** 2022-07-01

**Authors:** Fang-Ling Li, Wu-Chien Chien, Chi-Hsiang Chung, Chung-Yu Lai, Nian-Sheng Tzeng

**Affiliations:** 1 Department of Psychiatry, Tri-Service General Hospital Beitou Branch, National Defense Medical Center, Taipei City 112, Taiwan; fanglinlee@gmail.com; 2 Department of Psychiatry, Tri-Service General Hospital, National Defense Medical Center, Taipei City 114, Taiwan; 3 Department of Medical Research, Tri-Service General Hospital, School of Medicine, National Defense Medical Center, Taipei City 114, Taiwan; chienwu@mail.ndmctsgh.edu.tw (W.-C.C.); g694810042@mail.ndmctsgh.edu.tw (C.-H.C.); 4 School of Public Health, National Defense Medical Center, Taipei City 114, Taiwan; 5 Graduate Institute of Life Sciences, National Defense Medical Center, Taipei City 114, Taiwan; 6 Taiwanese Injury Prevention and Safety Promotion Association, Taipei City 114, Taiwan; 7 Graduate Institute of Aerospace and Undersea Medicine, National Defense Medical Center, Taipei City 114, Taiwan; 8 Student Counseling Center, National Defense Medical Center, Taipei City 114, Taiwan

**Keywords:** heat-related illness, psychiatric disorders, National Health Insurance Research Database

## Abstract

This study aimed to investigate the association between the heat-related illness (HRI) and the risk of developing psychiatric disorders. From 2000 to 2015, there were 3126 patients with newly diagnosed HRI selected from the National Health Insurance Research Database, along with 31,260 controls matched for gender and age. Fine and Gray’s analysis was used to compare the risk of psychiatric disorders during the 16 years of follow-up. Among the subjects, 523 of the HRI patients and 3619 of the control group (1774.18 vs. 1193.78 per 100,000 person-years) developed psychiatric disorders. Compared with non-HRI patients, the HRI ones had a 3.849-fold risk of being attacked by psychiatric disorders (95% CI: 3.632–4.369, *p* < 0.001) after adjusting for potential confounders. The sensitivity analysis revealed that the relationship between the HRI and the listed psychiatric disorders was determined by the exclusion of the first-year psychiatric events after the HRI. In spite of deleting the psychiatric diagnoses of the first five years, the HRI was still correlated with the development of psychiatric disorders with the exception of schizophreniform disorders, posttraumatic stress disorders, and acute stress disorder. Therefore, our findings concluded that the HRI could be a potential influence on the increased hazard of psychiatric disorders.

## 1. Introduction

With climate change, the damage caused by thermal energy has attracted more attention. About 42% of employees perceive that their workplaces are at risk of overheating and heat-related injury (HRI) in Taiwan [1]. From the studies in the USA [2] and Japan [3], HRI has caused around 500–600 deaths per year. HRI is also one of the most common causes of environment-related deaths [4]. Athletes, military personnel, and outdoor laborers are at greatest risk [5]. Military personnel are commonly recognized as one of the susceptible subgroups to HRI due to the characteristics of their mission: HRI often occurs in the general daily training and strenuous physical exertion; for example, heat stroke frequently caused unnecessary and preventable nontraumatic exertional deaths in the United States military [6]. In addition, there were 2163 and 464 personnel who were, respectively, attacked by heat-related illness and heat stroke in the U.S. Armed Forces in 2017, and the incidence rates were calculated as 1.41 and 0.38 personnel per 1000 person-years, respectively [5].

HRI includes heat stroke, heat syncope, heat cramps, and heat fatigue, which can lead to acute extensive, and severe physical impairment [7], and might result in multiple organ system problems and a long-term health impact [8]. HRI is associated with the increased risk of subsequent cardiovascular disease [9], chronic kidney disease [10], and brain injury with probable long-term neurological sequela [8,11]. One study in India revealed that nearly half the victims of HRI were with residual neurological sequela [12]. Furthermore, HRI was known to be harmful to the cognitive function [13]. One study showed that around 10–20% of heat stroke victims have had sequela of cognitive impairment [14]. Based on the harmful consequences of heat stress to the human brain, previous outcomes revealed that neurological disease often shares similar risk factors and pathogenesis to psychiatric diseases [15], so we hypothesized that HRI might cause the development of psychiatric disease. However, there are few studies that indicated if HRI could increase the risk of mental illness. Therefore, we conducted a cohort study by using Taiwan’s National Health Insurance Research Database (NHIRD), which included the representative medical records of the Taiwan population from 2000 to 2015, to clarify whether the HRI would be associated with the risk of developing psychiatric disorders, in a nationwide population.

## 2. Materials and Methods

### 2.1. Data Sources

The government of Taiwan launched the national health insurance (NHI) program since 1995, which is a single-payer and universal health coverage system. This program contained 97% of medical providers and covered approximately 23.67 million people in 2016 [16,17]. All the medical information was aggregated from the claims, and the diagnosis was recorded from the international classification of diseases, 9th revision, clinical modification (ICD-9-CM codes). The details of the NHIRD have been elaborated in previous studies [18,19,20,21,22,23].

In this study, we chose the longitudinal health insurance database (LHID), which consists of two-million cases, which contained a randomized, representative, sub-dataset selected from the NHIRD as the cohort. In addition, the traditional Chinese version of the ICD-9-CM was selected for the diagnosis, diseases coding, and the collection of data in the NHIRD [24]. We examined the accuracy of the data source, the NHIRD, which was validated as follows: First, the medical reviews to claims were conducted randomly by the NHI Administration (the frequency was one in every 100 outpatient visits and one in 20 in-patient admissions) to confirm the accuracy of the diagnoses [25]. Second, the diagnostic coding will receive further review and be confirmed in the medical institution by the licensed medical records technicians before the claim is reimbursed [26]. Third, in recent years, the accuracy and reliability of the NHIRD studies were greatly increased after the validations of the diagnosed codes or addresses of the unmeasured confounders via the methodologic approaches [17]. Therefore, the NHIRD was the effective tool to complete this study.

### 2.2. Study Design

A retrospective cohort study was organized to investigate the association of interest. Patients who sought medical help from hospitals due to the HRI during the period from 2000 to 2015 were selected from the LHID and categorized based on the ICD-9-CM codes as 992. The types of HRI contained heat stroke and other HRI such as heat syncope, heat cramps, heat exhaustion, heat edema, and other unspecified effects quantified as heat effects. The reasons and origins of the HRI included weather conditions, man-made conditions, and unspecified origins. The ICD codes for these diagnoses and conditions are as listed in Table S1.

To determine the risk level of psychiatric disorders among the HRI patients, a 1:10 age-, gender-, and index-year matched the non-HRI group. We excluded those patients who suffered from HRI before 2000 and psychiatric disorders before the tracking. The patients aged <20 were also excluded. All the psychiatric disorders were diagnosed by certified psychiatrists in Taiwan.

The ethical committee of the Institutional Review Board of the Tri-Service General Hospital reviewed and approved this study (TSGH IRB No. C202005160). Because this study was classified into the low-risk design, written informed consent for participation was not necessary in accordance with the national legislation and the institutional requirements

### 2.3. Variable Measures

In this study, the psychiatric disorders included were dementia, anxiety, depression, bipolar disorder, sleep disorders, and post-traumatic stress disorder/acute stress disorder (PTSD/ASD); psychotic disorders, including schizophrenia, schizophreniform disorder, and other psychotic disorders; substance-related disorders (SRD), including alcohol usage disorders and illicit drug usage disorders. In accordance with the criteria of ICD-9-CM codes, each patient diagnosed with a psychiatric disorder was required to have made at least three outpatient visits within one consecutive year in the study period [27,28].

The cofactors contained the years of age (20–49, 50–64, and 65 years), gender (male and female), geographical area of residence (north, center, south, and east Taiwan), urbanization level of residence (levels 1 to 4), and monthly income (in New Taiwan Dollars (TWD); <18,000, 18,000–34,999, and ≥35,000). The aforementioned comorbidities were evaluated by using the Charlson comorbidity index (CCI). ICD-9-CM codes were used as the fundamentals for the establishment of categories for comorbidities, each category was assigned to a single score, and CCI scores were combined, with 0 indicating no comorbidities and higher scores (1, 2, 3, 4) indicating higher comorbidity burdens [29,30].

### 2.4. Statistical Analyses

All data were managed and analyzed by the Statistical Product and Service Solutions (version 22.0, IBM Corp., Armonk, NY, USA). In the univariate test, the mean difference of continuous variables between the HRI and non-HRI groups was examined by the independent *t* test.

The chi-square test and Fisher exact test were used to determine the proportion differences of discrete factors between the two cohorts. The Kaplan–Meier method and log-rank test were used to estimate the cumulative incidence of psychiatric disorders in the two groups. In the multivariate test, Fine and Gray’s survival analysis was applied to calculate the risk of psychiatric disorders between the HRI cohort and the non-HRI groups. A two-tailed *p*-value less than 0.05 was considered as being of statistically significant difference.

## 3. Results

### 3.1. Description of Study Cohorts with and without HRI at the Baseline

Table 1 shows the characteristics of both cohorts, and the HRI cohort was predominantly male, mostly young to middle aged, and with annual insured premium costs at the baseline. The CCI scores in the with/without HRI group were 0.52 and 0.71, respectively. The majority of the HRI cases occurred in summer, but there was no significant difference between the seasons. In addition, the HRI group tended to live at the residence of urbanization of level 2 and level 4, and in the middle area of Taiwan, as well as seek help from regional and local hospitals.

### 3.2. Heat-Related Illness and the Risk of Psychiatric Disorders

Compared with the control group, Fine and Gray’s survival analysis revealed that the crude sub-distribution hazard ratio (sHR) for the risk of psychiatric disorders in the HRI cohort was 3.978 (95% CI: 3.632–4.369, *p* < 0.001). The adjusted sHR was 3.849 (95% CI: 3.632–4.369, *p* < 0.001) after adjusting for other confounders (Table 2). In addition, HRI patients with male gender; aged 50–64 and ≥65; with CCI_R groups of 1, 2, 3, and ≥4; with residence in the region of urbanization level 1; seeking medical help in the medical center or regional hospital tended to have an elevated risk of psychiatric disorders. In contrast, the HRI cohort with a monthly insured premium ≥ 35,000 and medical help-seeking in autumn was associated with a lower risk of psychiatric disorders.

### 3.3. Kaplan–Meier Model for the Cumulative Incidence of Psychiatric Disorders

**Figure 1:**
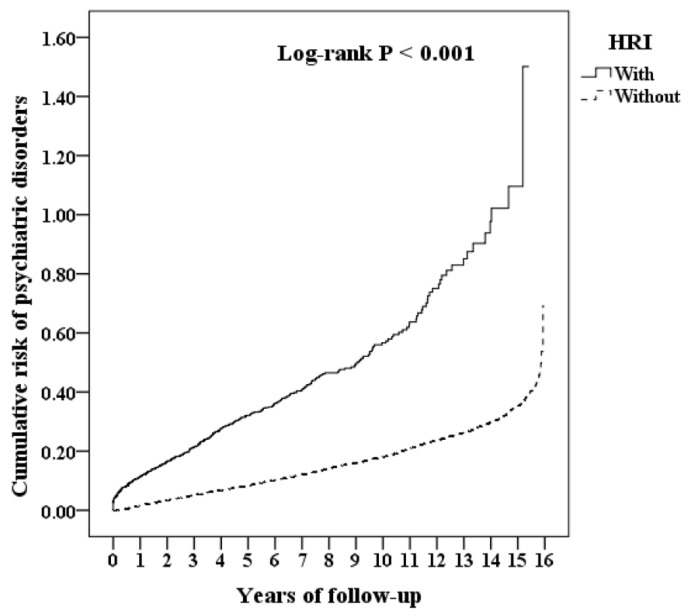
Kaplan–Meier for cumulative risk comparison of psychiatric disorders between the HRI and non-HRI groups.

During the whole study period, 523 of the 3126 HRI patients (1774.18 per 100,000 person-years) developed psychiatric disorders as compared to 3619 of the 31,260 patients in the control group (1193.78 per 100,000 person-years). Kaplan–Meier analysis indicated that the cumulative risk of psychiatric diseases at the HRI group was statistically and significantly higher than that of the non-HRI group during the 15 years (log-rank, *p* < 0.001, Figure 1).

### 3.4. Subgroup Analysis Stratified by the Different Characteristics

The subgroup analysis was repeatedly conducted based on the different classification of the characteristics. Our results declared that every single subgroup of HRI patients categorized by the characteristic variables was associated with a higher risk of psychiatric disorders, with the exceptions of the insured premium TWD ≥ 35,000 (Table 3).

### 3.5. Heat-Related Illnesses, the Situations, and the Association with Psychiatric Disorders

Former findings have indicated the relationship between the HRI and psychiatric disorders. In a further analysis, Table S2 shows the risk levels to the occurrence of psychiatric disorders at the different HRI types and situations. Compared with the non-HRI group, the adjusted sHRs of the ones with heat stroke and other HRIs were 3.657 (*p* < 0.001) and 3.933 (*p* < 0.001), respectively. Regardless of whether the sources of heat were from weather, man-made, or unspecific conditions, patients with HRI were all associated with the risk of a significant increment in psychiatric disorders.

### 3.6. Sensitivity Analysis

HRI was associated with the risk of overall psychiatric disorders, dementia, anxiety, depression, bipolar, sleep disorders, and PTSD/ASD; psychotic disorders, including schizophrenia and schizophreniform disorders; other psychotic disorders; alcohol or other illicit usage disorders. We attempted to validate the accuracy of the connection between the HRI and psychiatric disorders. Two sensitivity analyses were conducted by the exclusion of the first 1-year and 5-year patients where the psychiatric disorders occurred after the HRI. As shown in Table 4, the adjusted overall sHRs were 2.804 (*p* < 0.001) and 3.235 (*p* < 0.001), respectively, after not enrolling the psychiatric diagnoses of the first 1 year and 5 years after the HRI. In addition, most of the psychiatric disorders were also related to the HRI attack, with the exception of schizophreniform disorders and PTSD/ASD (Table 4). Among the HRI patients, the mean time to the development of psychiatric disorders was 2.92 ± 3.43 years, much less than 6.48 ± 4.34 years in comparison to the non-HRI controls (Table S3).

## 4. Discussion

To the best of our knowledge, this study is the first, as the pilot study focused on the topic of the relationship between HRI and the risk of psychiatric disorders, in a nationwide, population-based cohort. The main results showed that the patients diagnosed with HRI had more chances of developing psychiatric disorders when compared with the non-HRI group due to the heat stress that might damage the human brain. Heat-related injury did increase the risk of different types of psychiatric disorders, while there were consistent results determined in the sensitivity analysis. Furthermore, we also examined the different situations of HRI occurrence, and the influence still existed.

In this large-scale retrospective cohort study, there are also several noteworthy and valuable findings to be discussed with the previous research. First, we discovered that the patients with HRI had about a fourfold risk of psychiatric disorders, and the subgroup analysis still revealed the conformity of the HRI cohort in the stratified levels of the major covariates. Furthermore, the probability of psychiatric disorders increased all of the different HRI types and origins of heat. Despite several previous studies on the issue of extreme heat with mental health problems [31,32], this current study was the first work to quantify the risk of the subsequent development of psychiatric illness after the HRI took place. 

Second, after excluding the diagnosis of psychiatric disorders for the first five years after HRI was identified, the HRI cohort was still associated with the elevated risk for developing overall psychiatric and individual psychiatric disorders, with the exception of PTSD/ASD and schizophreniform disorders. We also found that the risk of development of PSTD was not beyond the first five years. Similarly, studies on the topic about severe or extreme injuries or disease have also revealed that the survivors would have developed psychiatric disorders over several years after the exposure to these events [19,33,34,35,36]. Therefore, it could be important for the clinicians who care for the HRI survivors to monitor their mental health problems, such as PTSD/ASD, in the first three to four years after the HRI diagnosis.

Third, men with a history of HRI have a 10% higher risk of psychiatric disorders than women in our data. Previous studies have shown that the incidence of HRI in men is already higher than in women [37]; Sugg et al., (2016) demonstrated that the HRI rates were nearly four times greater for the male population in America [38]. In Asian areas, Na et al., (2013) also revealed that the incidence of heat-related illnesses was higher in men than women in Korea [39]. In line with former reports, our work also showed that the male group accounted for almost 80% of the HRI group. This means that we should pay more attention to the harmful effects of heat to the male group because male patients seemed to be more vulnerable to developing psychiatric disorders after heat injury than females.

Fourth, in the present study, some socioeconomic factors, such as the urbanization, hospital levels, and monthly insured premiums, were associated with varied risks of psychiatric disorders. We assumed that the HRI group might possibly face a relatively harsh social and environmental condition. For example, soldiers are often trained to tolerate the relatively unfriendly environment in the battlefield, which is overheated and without proper cooling equipment. In addition, they are often subjected to enormous work stress, not only from training but also from the other duties that could make them susceptible to mental illness [40]. The same scenario should be observed at the outdoor worker group. In accordance with data from Taiwan, over two-thirds of the employees who often complain about overheating in the workplace have a low level of education [1]. Furthermore, low socioeconomic status might be associated with an increased risk of developing mental illness [41].

However, HRI was not significantly associated with schizophrenia and PTSD/ASD after excluding psychiatric diagnoses in the previous five-year data. There are several potential explanations: (1) there are only a small number of cases to lower the statistical power to distinguish the difference; (2) due to the rapid intervention and treatment to the HRI in Taiwan, most cases could have a good prognosis [5] and have less opportunity to progress to mental disability. Thus, the magnitude of the psychological impact of trauma was decreased.

### 4.1. Potential Underlying Mechanisms

The onset of psychiatric disorders is often driven by biopsychosocial factors. A previous study of an animal model demonstrated that “hyperthermia” is relative to the profound brain dysfunction (e.g., blood–brain barrier disruption and brain edema formation), which leads to severe cognitive, sensory, and motor dysfunction [42]. Those neurological deficits mentioned above might have similar pathogenetic pathways of psychiatric disorders [15]. Our findings confirmed that the HRI had an influence on the elevated risk of psychiatric disorder development. However, patients, after having recovered from the HRI, could be again exposed to the relatively poor and difficult environmental stress during their work. The condition might possibly be one of the enhancing factors for the onset of mental illness [43], further increasing the chance of mental illness development. Therefore, a specific mechanism is still needed for a further extended study for clarity and proof.

### 4.2. Strengths

There are several strengths in this study: First, we extracted the longitudinal health insurance database, which contains a prodigious number of samples in this study. Second, there were some studies that considered the relationship between the incidence of HRI and preexisting psychiatric disorder; however, there is still no study that has discussed the association between HRI and the risk of following psychiatric disorders, the mechanism of which is also still unclear. Next, this was a long-term follow-up study of 16 years. We attempted to clarify as to whether there is a relationship between the development of psychiatric disorders and the experiment of HRI. Additionally, we conducted the competing risk model to adjust the all-cause mortality, to see whether there was a possibility that existed of a survival bias that may interfere with the outcome; however, the risk between HRI and the following psychiatric disorders still remained.

### 4.3. Limitations

This study had some inherent limitations. First, in the NHIRD, there was a lack of information on the severity and laboratory data among these HRI patients; in addition, the genetic, environmental, and psychosocial factors were not recorded in the database, which is similar to previous research using the NHIRD. Second, if the patients had consulted for psychiatric situations without official records for their personal concern of privacy, it may lead to ascertainment bias in the clinical setting. Third, there were only a few numbers of PTSD/ASD, when schizophreniform disorder occurred within five years after HRI; however, based on the traumatized characteristic of a heat-related event, the stress and trauma-related effect still needs further attention. Finally, there were no details of side information from the HRI patients’ family members and the health staff who looked after them in the NHIRD. Further studies to explore the post-HRI psychiatric comorbidity is needed in any future follow-up.

## 5. Conclusions

In conclusion, the findings of this long-term follow-up study indicate the existence of an association between HRI and the increasing risk of psychiatric disorders. This strong evidence should serve as a reminder that the physicians should pay more attention to the psychiatric comorbidity of the patients with HRI.

## Figures and Tables

**Table 1:** Characteristics and comparison of study patients between the with and without HRI at the baseline.

Variables	Total *(n* = 34,386) *n* (%)	With HRI (*n* = 3126) *n* (%)	Without HRI (*n* = 31,260) *n* (%)	*p* Value
Gender				0.999
Male	27,687 (80.52)	2517 (80.52)	25,170 (80.52)	
Female	6699 (19.48)	609 (19.48)	6090 (19.48)	
Age (years)	45.95 ± 17.20	45.57 ± 16.68	45.99 ± 17.25	0.193
Age groups (years)				0.999
20–49	20,548 (59.76)	1868 (59.76)	18,680 (59.76)	
50–64	6413 (18.65)	583 (18.65)	5830 (18.65)	
≥65	7425 (21.59)	675 (21.59)	6750 (21.59)	
Insured premium (TWD)				0.084
<8000	33,753 (98.16)	3065 (98.05)	30,688 (98.17)	
18,000–34,999	456 (1.33)	51 (1.63)	405 (1.30)	
≥35,000	177 (0.51)	10 (0.32)	167 (0.53)	
CCI_R	0.70 ± 1.67	0.52 ± 1.42	0.71 ± 1.69	<0.001
CCI_R groups				<0.001
0	23,884 (69.46)	2297 (73.48)	21,587 (69.06)	
1	5475 (15.92)	494 (15.80)	4981 (15.93)	
2	2026 (5.89)	177 (5.66)	1849 (5.91)	
3	1534 (4.46)	87 (2.78)	1447 (4.63)	
≥4	1467 (4.27)	71 (2.27)	1396 (4.47)	
Season				0.999
Spring (March–May)	5412 (15.74)	492 (15.74)	4920 (15.74)	
Summer (June–August)	21,758 (63.28)	1978 (63.28)	19,780 (63.28)	
Autumn (September–November)	5159 (15.00)	469 (15.00)	4690 (15.00)	
Winter (December–February)	2057 (5.98)	187 (5.98)	1870 (5.98)	
Location				<0.001
Northern Taiwan	13,045 (37.94)	692 (22.14)	12,353 (39.52)	
Middle Taiwan	9919 (28.85)	1175 (37.59)	8744 (27.97)	
Southern Taiwan	8915 (25.93)	773 (24.73)	8142 (26.05)	
Eastern Taiwan	2319 (6.74)	443 (14.17)	1876 (6.00)	
Outlets islands	188 (0.55)	43 (1.38)	145 (0.46)	
Urbanization level				<0.001
1 (The highest)	11,149 (32.42)	481 (15.39)	10,668 (34.13)	
2	14,205 (41.31)	1361 (43.54)	12,844 (41.09)	
3	3111 (9.05)	413 (13.21)	2698 (8.63)	
4 (The lowest)	5921 (17.22)	871 (27.86)	5050 (16.15)	
Level of care				<0.001
Hospital center	9978 (29.02)	394 (12.60)	9584 (30.66)	
Regional hospital	10,668 (31.02)	1274 (40.75)	9394 (30.05)	
Local hospital	13,740 (39.96)	1458 (46.64)	12,282 (39.29)	

HRI: heat-related illnesses; TWD: New Taiwan Dollars; CCI_R: Charlson comorbidity index revised.

**Table 2:** Factors of psychiatric disorders by using Cox regression with/without Fine and Gray’s competing risk model.

Variables	No Competing Risk in the Model			Competing Risk in the Model		
	Adjusted HR	95% CI	*p* Value	Adjusted HR	95% CI	*p* Value
HRI						
Without	Reference			Reference		
With	3.748	3.412–4.117	<0.001	3.849	3.522–4.206	<0.001
Gender						
Male	1.101	1.017–1.192	0.017	1.128	1.043–1.221	0.003
Female	Reference			Reference		
Age groups (years)						
20–49	Reference					
50–64	1.506	1.465–1.551	<0.001	1.517	1.475–1.563	<0.001
≥65	1.649	1.602–1.701	<0.001	1.733	1.680–1.791	<0.001
Insured premium (TWD)						
<18,000	Reference			Reference		
18,000–34,999	0.827	0.641–1.067	0.145	0.799	0.619–1.031	0.085
≥35,000	0.212	0.079–0.565	0.002	0.209	0.078–0.556	0.002
CCI_R groups						
0	Reference			Reference		
1	1.474	1.365–1.593	<0.001	1.520	1.407–1.642	<0.001
2	1.633	1.474–1.808	<0.001	1.756	1.585–1.945	<0.001
3	1.223	1.069–1.399	0.003	1.391	1.216–1.592	<0.001
≥4	1.002	0.889–1.130	0.973	1.331	1.180–1.503	<0.001
Season						
Spring (March–May)	Reference			Reference		
Summer (June–August)	0.998	0.816–1.088	0.970	0.998	0.916–1.087	0.959
Autumn (September–November)	0.873	0.800–0.952	0.002	0.865	0.793–0.944	0.001
Winter (December–February)	0.976	0.894–1.065	0.584	0.982	0.899–1.071	0.679
Urbanization level						
1 (The highest)	1.221	1.107–1.347	<0.001	1.179	1.069–1.301	<0.001
2	1.036	0.914–1.184	0.903	1.023	0.903–1.160	0.721
3	1.005	0.929–1.097	0.704	1.004	0.927–1.088	0.923
4 (The lowest)	Reference			Reference		
Level of care						
Hospital center	1.713	1.557–1.885	<0.001	1.752	1.592–1.929	<0.001
Regional hospital	1.222	1.124–1.329	<0.001	1.225	1.126–1.332	<0.001
Local hospital	Reference			Reference		<0.001

HRI: heat-related illnesses; TWD: New Taiwan Dollars; CCI_R: Charlson comorbidity index revised; HR: hazard ratio; CI: confidence interval.

**Table 3:** Factors of psychiatric disorders stratified by variables listed in the table by using Cox regression with/without Fine and Gray’s competing risk model.

Stratified Variables	With HRI			Without HRI (Reference)			Competing Risk in the Model		
	Events	PYs	Rate (Per 10^5^ PYs)	Events	PYs	Rate (Per 10^5^ PYs)	Adjusted sHR	95% CI	*p* Value
Total	523	29,478.47	1774.18	3619	303,153.95	1193.78	3.849	3.522–4.206	<0.001
Gender									
Male	398	21,718.98	1832.50	2845	240,747.97	1181.73	4.016	3.675–4.389	<0.001
Female	125	7759.48	1610.93	774	62,405.99	1240.27	3.364	3.078–3.676	<0.001
Age groups (years)									
20–49	205	10,686.70	1918.27	1457	93,914.94	1551.40	3.202	2.930–3.499	<0.001
50–64	117	6407.14	1826.09	1046	89,618.76	1167.17	4.052	3.708–4.428	<0.001
≥65	201	12,384.63	1622.98	1116	119,620.24	932.95	4.505	4.123–4.923	<0.001
Insured premium (TWD)									
<18,000	517	28,817.05	1794.08	3568	296,634.58	1202.83	3.863	3.535–4.221	<0.001
18,000–34,999	6	627.18	956.66	48	4950.82	969.54	2.555	2.338–2.792	<0.001
≥35,000	0	34.24	0.00	3	1568.56	191.26	-	-	0.995
CCI_R groups									
0	190	15,559.82	1221.09	1660	153,252.94	1083.18	2.920	2.672–3.190	<0.001
1	158	6051.41	2610.96	997	66,703.94	1494.66	4.524	4.140–4.944	<0.001
2	101	2920.17	3458.70	457	29,081.72	1571.43	5.700	5.216–6.229	<0.001
3	25	1679.63	1488.42	186	20,875.25	891.01	4.326	3.959–4.728	<0.001
≥4	49	3267.44	1499.65	319	33,240.11	959.68	4.047	3.703–4.422	<0.001
Season									
Spring (March–May)	155	6795.83	2280.81	901	72,017.75	1251.08	4.722	4.320–5.159	<0.001
Summer (June–August)	142	6900.78	2057.74	931	77,668.50	1198.68	4.446	4.068–4.858	<0.001
Autumn (September–November)	103	8563.55	1202.77	907	81,760.81	1109.33	2.808	2.569–3.068	<0.001
Winter (December–February)	123	7218.31	1704.00	880	71,706.88	1227.22	3.596	3.291–3.930	<0.001
Urbanization level									
1 (The highest)	112	6610.42	1694.30	924	91,033.70	1015.01	4.323	3.956–4.724	<0.001
2	222	14,550.52	1525.72	1329	134,057.66	991.36	3.986	3.647–4.356	<0.001
3	50	2772.35	1803.53	331	24,698.28	1340.17	3.485	3.189–3.809	<0.001
4 (The lowest)	139	5545.18	2506.68	1035	53,364.31	1939.50	3.347	3.063–3.658	<0.001
Level of care									
Hospital center	102	7590.10	1343.86	894	101,204.83	883.36	3.940	3.605–4.305	<0.001
Regional hospital	238	14,836.84	1604.12	1511	138,305.48	1092.51	3.803	3.480–4.155	<0.001
Local hospital	183	7051.53	2595.18	1214	63,643.64	1907.50	3.524	3.224–3.850	<0.001

TWD: New Taiwan Dollars; CCI_R: Charlson comorbidity index revised; HRI: heat-related illnesses; PYs: person-years; sHR: subdivision hazard ratio; CI: confidence interval; Competing variables: all-cause mortality.

**Table 4:** Sensitivity test for factors of psychiatric disorders subgroup by using Cox regression with/without Fine and Gray’s competing risk model.

Sensitivity Test	Psychiatric Disorders Subgroup	With HRI			Without HRI (Reference)			Competing Risk in the Model		
		Events	PYs	Rate (Per 10^5^ PYs)	Events	PYs	Rate (Per 10^5^ PYs)	Adjusted sHR	95% CI	*p* Value
Overall	Overall	523	29,478.47	1774.18	3619	303,153.95	1193.78	3.849	3.522–4.206	<0.001
	Dementia	87	29,478.47	295.13	729	303,153.95	240.47	3.179	2.908–3.473	<0.001
	Anxiety	81	29,478.47	274.78	498	303,153.95	164.27	4.332	3.964–4.734	<0.001
	Depression	88	29,478.47	298.52	535	303,153.95	176.48	4.381	4.009–4.787	<0.001
	Bipolar	16	29,478.47	54.28	78	303,153.95	25.73	5.463	4.999–5.970	<0.001
	Sleep disorders	114	29,478.47	386.72	806	303,153.95	265.87	3.767	3.447–4.116	<0.001
	PTSD/ASD	8	29,478.47	27.14	10	303,153.95	3.30	21.307	19.497–23.283	<0.001
	Psychotic disorders	57	29,478.47	193.36	419	303,153.95	138.21	3.623	3.315–3.959	<0.001
	Schizophrenia	35	29,478.47	118.73	234	303,153.95	77.19	3.984	3.645–4.353	<0.001
	Schizophreniform	3	29,478.47	10.18	3	303,153.95	0.99	26.634	24.371–29.104	<0.001
	Other psychotic disorders	17	29,478.47	57.67	122	303,153.95	40.24	3.711	3.396–4.056	<0.001
	SRD	143	29,478.47	485.10	904	303,153.95	298.20	4.213	3.855–4.604	<0.001
	AUD	123	29,478.47	417.25	818	303,153.95	269.83	4.005	3.665–4.376	<0.001
	IDUD	22	29,478.47	74.63	97	303,153.95	32.00	6.041	5.527–6.601	<0.001
In the first 1 years excluded	Overall	303	25,760.55	1176.22	3270	301,005.38	1086.36	2.804	2.566–3.064	<0.001
	Dementia	62	25,760.55	240.68	697	301,005.38	231.56	2.692	2.463–2.942	<0.001
	Anxiety	43	25,760.55	166.92	456	301,005.38	151.49	2.854	2.611–3.118	<0.001
	Depression	44	25,760.55	170.80	495	301,005.38	164.45	2.690	2.461–2.939	<0.001
	Bipolar	8	25,760.55	31.06	69	301,005.38	22.92	3.509	3.211–3.834	<0.001
	Sleep disorders	55	25,760.55	213.50	765	301,005.38	254.15	2.176	1.991–2.377	<0.001
	PTSD/ASD	2	25,760.55	7.76	9	301,005.38	2.99	6.725	6.154–7.349	<0.001
	Psychotic disorders	31	25,760.55	120.34	325	301,005.38	107.97	2.887	2.641–3.154	<0.001
	Schizophrenia	20	25,760.55	77.64	218	301,005.38	72.42	2.776	2.540–3.034	<0.001
	Schizophreniform	1	25,760.55	3.88	3	301,005.38	1.00	10.087	9.230–11.023	<0.001
	Other psychotic disorders	8	25,760.55	31.06	106	301,005.38	35.22	2.284	2.090–2.496	<0.001
	SRD	96	25,760.55	372.66	781	301,005.38	259.46	3.720	3.404–4.065	<0.001
	AUD	81	25,760.55	314.43	706	301,005.38	234.55	3.472	3.177–3.794	<0.001
	IDUD	17	25,760.55	65.99	85	301,005.38	28.24	6.052	5.538–6.614	<0.001
In the first 5 years excluded	Overall	113	11,982.97	943.00	2052	271,841.51	754.85	3.235	2.961–3.535	<0.001
	Dementia	19	11,982.97	158.56	507	271,841.51	186.51	2.202	2.015–2.406	<0.001
	Anxiety	17	11,982.97	141.87	250	271,841.51	91.97	3.995	3.656–4.366	<0.001
	Depression	16	11,982.97	133.52	321	271,841.51	118.08	2.928	2.680–3.200	<0.001
	Bipolar	2	11,982.97	16.69	44	271,841.51	16.19	2.671	2.444–2.918	<0.001
	Sleep disorders	27	11,982.97	225.32	503	271,841.51	185.03	3.154	2.886–3.446	<0.001
	PTSD/ASD	0	11,982.97	0.00	8	271,841.51	2.94	0.000	-	0.993
	Psychotic disorders	7	11,982.97	58.42	198	271,841.51	72.84	2.077	1.901–2.270	<0.001
	Schizophrenia	4	11,982.97	33.38	142	271,841.51	52.24	1.655	1.514–1.809	<0.001
	Schizophreniform	0	11,982.97	0.00	2	271,841.51	0.74	0.000	-	0.995
	Other psychotic disorders	2	11,982.97	16.69	56	271,841.51	20.60	2.098	1.920–2.293	<0.001
	SRD	38	11,982.97	317.12	423	271,841.51	155.61	5.278	4.830–5.768	<0.001
	AUD	31	11,982.97	258.70	373	271,841.51	137.21	4.883	4.468–5.336	<0.001
	IDUD	8	11,982.97	66.76	56	271,841.51	20.60	8.393	7.680–9.172	<0.001

HRI: heat-related illnesses; PYs: person-years; sHR: subdivision hazard ratio; CI: confidence interval; Competing variables: all-cause mortality; PTSD/ASD: posttraumatic stress disorder/acute stress disorder; SRD: Substance-related disorders; AUD: Alcohol use disorder; IDUD: Illicit drug use disorder.

## Data Availability

Data are from the National Health Institute Research Database, which is available to researchers in Taiwan and has been extensively used in epidemiologic studies. Use is allowed for academic purpose only after proof. Thus, the data cannot be made publicly available.

## Supplementary Materials

The following are available online at https://www.mdpi.com/article/10.3390/ijerph19138087/s1, Table S1: Abbreviation, ICD-9-CM, and definition. Table S2: Factors of psychiatric disorders stratified by HRI subgroup by using Cox regression with/without Fine and Gray’s competing risk model. Table S3: Years to psychiatric disorders.
