# Supplementary material for: Real-World Evidence for the Association between Heat-Related Illness and the Risk of Psychiatric Disorders in Taiwan
Source: Int J Environ Res Public Health. 2022 Jul 1;19(13):8087. doi: 10.3390/ijerph19138087 (PMC9265553; doi:10.3390/ijerph19138087)
Supplement: Supplementary file 1 [file ijerph-19-08087-s001.zip › ijerph-1766549-supplementary.pdf]

Table S1. Abbreviation, ICD-9-CM, and definition.

Variables	Abbreviation	ICD-9-CM/ definition
Study cohort: Heat related illness	HRI	992
Heat stroke		992.0
Other HRI		992.1-992.9
Heat syncope		992.1
Heat cramps		992.2
Heat exhaustion		992.3-992.6
Heat edema		992.7
Other specified and unspecified heat effects		992.8-992.9
Weather conditions		E900.0
Man-made conditions		E900.1
Unspecified origins		E900.9
Events: Psychiatric disorders		≧3 OPD/ER visits or IPD
Dementia		290.0, 290.10-290.13, 290.20-290.21, 290.3, 290.41-290.43, 290.8-290.9, 331.0
Anxiety disorders	Anxiety	300
Depressive disorders	Depression	296.2-296.3, 300.4, 311
Bipolar disorders	Bipolar	296.0, 296.4-296.8
Sleep disorders		307.4, 780.5
Posttraumatic stress disorder/ acute stress disorder	PTSD/ASD	308, 309.81
Psychotic disorders		295, 297-298
Schizophrenia		295 except 295.4
Schizophreniform disorder	Schizophreniform	295.4
Other psychotic disorders		297-298
Substance-related disorders	SRD	291-292, 303.0, 303.9, 304-305
Alcohol use disorder	AUD	291, 303.0, 303.9, 305.0
Illicit drug use disorder	IDUD	292, 304-305 except 305.0
Comorbidities: Charlson comorbidity index revised	CCI_R	CCI removed dementia
Medical visits		
Outpatient department	OPD	
Emergency room	ER	
Inpatient department	IPD	

ICD-9-CM: International Classification of Diseases, Ninth Revision, Clinical Modification.

Table S2. Factors of psychiatric disorders stratified by HRI subgroup by using Cox regression with / without Fine & Gray's competing risk model.

HRI subgroup	Populations	Events	PYs	Rate (per 10^5 PYs)	Competing risk in the model		
					Adjusted sHR	95% CI	*P* value
**Without HRI**	**31,260**	**3,619**	**303,153.95**	1,193.78	Reference		
With HRI	3,126	523	29,478.47	1,774.18	3.849	3.522-4.206	<0.001
Heat stroke	1,503	242	13,603.27	1,778.98	3.657	3.205 -4.173	<0.001
Other HRI	1,623	281	15,875.20	1,770.06	3.933	3.482-4.452	<0.001
Weather conditions	162	25	1,527.63	1,636.52	3.550	3.250-3.888	<0.001
Man-made conditions	151	23	1,423.96	1,615.21	3.501	3.207-3.830	<0.001
Unspecified origins	2,813	475	26,526.88	1,790.64	3.889	3.557-4.248	<0.001

HRI: heat-related illnesses; PYs: person-years; sHR: subdivision hazard ratio; CI: confidence interval; Competing variables: all-caused mortality.

Table S3. Years to psychiatric disorders

HRI	Min	Median	Max	Mean ± SD
With	0.01	1.66	15.19	2.92 ± 3.43
Without	0.01	5.91	15.93	6.48 ± 4.34
Total	0.01	5.35	15.93	6.03 ± 4.39

HRI: heat-related illnesses; SD: standard deviation.
